# Financial Stress Interacts With *CLOCK* Gene to Affect Migraine

**DOI:** 10.3389/fnbeh.2019.00284

**Published:** 2020-01-24

**Authors:** Daniel Baksa, Xenia Gonda, Nora Eszlari, Peter Petschner, Veronika Acs, Lajos Kalmar, J. F. William Deakin, Gyorgy Bagdy, Gabriella Juhasz

**Affiliations:** ^1^SE-NAP2 Genetic Brain Imaging Migraine Research Group, Hungarian Brain Research Program, Semmelweis University, Budapest, Hungary; ^2^Department of Pharmacodynamics, Faculty of Pharmacy, Semmelweis University, Budapest, Hungary; ^3^NAP-2-SE New Antidepressant Target Research Group, Hungarian Brain Research Program, Semmelweis University, Budapest, Hungary; ^4^Department of Psychiatry and Psychotherapy, Semmelweis University, Budapest, Hungary; ^5^MTA-SE Neuropsychopharmacology and Neurochemistry Research Group, Hungarian Academy of Sciences, Semmelweis University, Budapest, Hungary; ^6^Research Centre for Natural Sciences, Institute of Enzymology, Budapest, Hungary; ^7^Department of Veterinary Medicine, University of Cambridge, Cambridge, United Kingdom; ^8^Neuroscience and Psychiatry Unit, The University of Manchester and Manchester Academic Health Sciences Centre, Manchester, United Kingdom

**Keywords:** migraine, circadian rhythms, circadian locomotor output cycles kaput, financial hardship, chronic stress, gene × environment interaction

## Abstract

Previous studies suggested that both maladaptive stress response and circadian dysregulation might have a role in the background of migraine. However, effects of circadian genes on migraine have not been tested yet. In the present study, we investigated the main effect of rs10462028 of the circadian locomotor output cycles kaput (*CLOCK*) gene and its interaction with different stress factors on migraine. In our cross-sectional study 2,157 subjects recruited from Manchester and Budapest completed the ID-Migraine questionnaire to detect migraine type headaches (migraineID). Additional stress factors were assessed by a shortened version of the Childhood Trauma Questionnaire, the List of Threatening Experiences questionnaire, and a validated questionnaire to identify financial difficulties. Rs10462028 showed no main genetic effect on migraineID. However, chronic stress indexed by financial difficulties showed a significant interaction effect with rs10462028 (*p* = 0.006 in recessive model) on migraineID. This result remained significant after correction for lifetime bipolar and unipolar depression and was replicated in both subsamples, although only a trend effect was reached after Bonferroni-correction, which is the strictest correction not considering interdependences. Childhood adversity (CHA) and Recent negative life events (RLE) showed no significant gene × stress interaction with rs10462028. In addition, *in silico* analysis demonstrated that the genetic region tagged by rs10462028 alters the binding of several miRNAs. Our exploratory study suggests that variations in the *CLOCK* gene, with moderating effect on gene function through miRNA binding, in interaction with financial difficulties might influence the risk of migraine-type headaches. Thus, financial hardship as a chronic stress factor may affect migraine through altering circadian rhythms.

## Introduction

Migraine is a debilitating disorder affecting approximately one billion people worldwide (Gormley et al., 2016). Despite extensive research, its pathomechanism is still questionable. Migraine is heritable with estimated values of 0.34–0.57 (Chasman et al., 2016) reflecting an important role of environmental factors, also. A large number of genes of small effects appear to be involved in migraine (Anttila et al., 2013; Gormley et al., 2016), and their interactions with environmental factors have also been suggested (Sauro and Becker, 2009; Juhasz et al., 2017) but there is a significant lack of gene × environment interaction studies of migraine. As our knowledge about the pathogenesis of migraine accumulates, we may still overlook important contributing factors. One of these somewhat unacknowledged components might be circadian rhythmicity.

Circadian rhythms regulate several important physiological parameters (Johnston, 2014) enabling the organism to anticipate and adapt to environmental changes as a form of predictive homeostatis (Moore-Ede, 1986). Circadian dysregulation has been connected previously to various diseases (Hansen et al., 2011), including depression (Kishi et al., 2009) which shows a strong comorbidity with migraine (Breslau et al., 2003).

Several studies revealed circadian periodicity of migraine attack onset (van Oosterhout et al., 2018), but the underlying mechanism is not understood yet (Gori et al., 2012). Sleeping problems are also regular among migraineurs (Rains, 2018). Melatonin also might have a role in the pathophysiology of migraine, for example through the hypothalamic output of melatonin cycle influencing the trigeminal nucleus caudalis, a known component of migraine pathophysiology (Vogler et al., 2006). Related investigations mostly show lower levels of melatonin among migraineurs vs. controls (Vogler et al., 2006). A recent study (van Oosterhout et al., 2018) suggested a different setting of the circadian pacemaker among migraineurs: migraineurs compared to headache-free controls declared to be more affected by changes in circadian rhythm and more prone to have early or late chronotypes. This suggestion may be supported by genetic data: in two families with advanced sleep phase syndrome, migraine associated with a mutation in the casein kinase I*δ* gene (*CK1δ*) showing phosphorylating effect on Per2 circadian protein (Brennan et al., 2013).

All these data together suggest a possible connection between circadian rhythms and migraine. However, the connection between (common) migraine headache and circadian genes has not been investigated yet.

**Figure 1:**
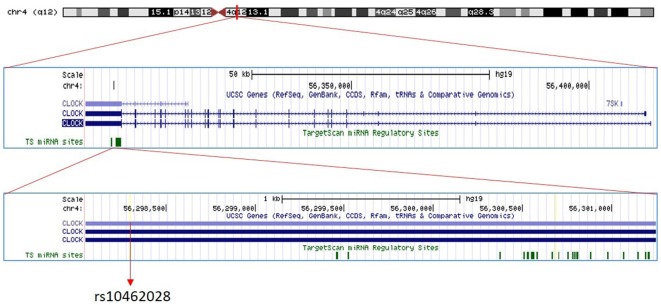
The position of *circadian locomotor output cycles kaput (CLOCK)* rs10462028. The position of rs10462028 on *CLOCK* gene which is located on chromosome four is shown. Created with UCSC Genome Browser (https://genome.ucsc.edu/).

The circadian pacemaker’s (suprachiasmatic nucleus—SCN) 24-h time-keeping capacity arises from a transcriptional/post-translation feedback loop with rhythmic expression of circadian genes (Hansen et al., 2011). One of these primary genes is circadian locomotor output cycles kaput gene (*CLOCK*) working as a transcriptional activator in the circadian clock mechanism. For our investigation we selected rs10462028 of *CLOCK* (Figure 1), which has been used as a tagSNP of *CLOCK* in a previous study showing its association with bipolar disorder (Soria et al., 2010)—a comorbid disease of migraine with overlapping genetic factors (Oedegaard et al., 2010).

Recent meta-analyses of genome-wide association studies (GWASs) for migraine (Anttila et al., 2013; Gormley et al., 2016) have not identified *CLOCK* as a susceptibility gene for migraine. However, besides genetic factors, environmental factors are important elements to address in migraine research, especially stress: as the disease has been connected to several stressors, including various forms of childhood abuse (Tietjen, 2016), recent negative life events (RLE; Juhasz et al., 2017), and low socioeconomic status (Stewart et al., 2013). A recent review (Koch et al., 2017) also discusses the connection between circadian rhythms and stress which is based on the interaction of the hypothalamic-pituitary-adrenal axis and autonomic nervous system, both playing roles in stress-related regulation and having strong circadian inputs.

A review (Sauro and Becker, 2009) collected multiple roles of stress in migraine. Stress is the factor most often listed by migraineurs as a trigger of their attacks. It can amplify attack intensity and duration and might be a risk factor for migraine chronification. Stress may play an important role in migraine comorbidities: for example, a prospective longitudinal study confirmed the known bidirectional connection of migraine and depression, but this significant association disappeared after controlling for the measured stress factors (Swanson et al., 2013). This strong connection of migraine and stress can be explained by the concept of maladaptive stress response among migraineurs: repeated stress leads to allostatic load causing alterations in normal homeostatic processes, failure in habituation, and closing of the stress response (Borsook et al., 2012). The “migraine brain” shows an enhanced perception of environmental stimuli (for a review see Schwedt and Chong, 2015) and structural and functional alterations in many brain regions including the ones that are directly involved in stress response (for example: amygdala, hypothalamus, hippocampus, prefrontal cortex; Borsook et al., 2012). These data confirm the need to include stress factors in migraine studies.

To further investigate the role of circadian rhythm in migraine, we tested in our exploratory study the effect of rs10462028 on migraine in interaction with three forms of stress that has been previously connected to migraine: childhood adversity (CHA), RLE, and low socioeconomic status in the form of financial difficulties.

## Materials and Methods

### Subjects

Two-thousand one-hundred and fifty-seven subjects were recruited through general practices and advertisements from Greater Manchester, UK (*n* = 1,277) and Budapest, Hungary (*n* = 880; aged between 18 and 60). The study was held under the aegis of NewMood (New Molecules in Mood Disorders, 2004-2009), an EU funded research program about pathomechanism of depression and related conditions. Our study was approved by the local Ethics Committees (Scientific and Research Ethics Committee of the Medical Research Council, Budapest, Hungary, ad.225/KO/2005; ad.323-60/2005-1018EKU and ad.226/KO/2005; ad.323-61/2005-1018 EKU; North Manchester Local Research Ethics Committee, Manchester, UK REC reference number: 05/Q1406/26) and was carried out in accordance with the Declaration of Helsinki. All participants administered written informed consent to the study. In the present investigation, we selected participants who were of European white origin, completed the questionnaires, and consented to DNA analysis. In every analysis we included subjects showing all the necessary data for the investigated variables. The total subject number was reached after screening for the following data: ethnicity, gender, age, migraine status, and rs10462028 genotype. Further details of the recruitment strategies and response rates can be found in our earlier publications (Juhasz et al., 2009; Lazary et al., 2009).

### Questionnaires

Brief standard questionnaires were administered for the study (English and Hungarian versions, respectively). The background questionnaire assembled information about socio-demographic data, personal and family psychiatric history. Gender, age, ethnicity, and reported lifetime bipolar disorder (MANIC) and lifetime depression (DEPR) data were derived from this validated questionnaire (Juhasz et al., 2011).

The ID-migraine questionnaire was used to measure migraine (migraineID). It is a validated screening tool for migraine (Lipton et al., 2003), which includes three items of the main migraine symptoms: nausea, photophobia, and disability (experienced in the past 3 months). In the present study, we defined migraineID as YES answers to two or three migraine symptom questions—this criterion has 0.93 positive predictive value of having migraine based on Lipton et al. (2003).

The following tools were applied to capture different stress factors. A shortened version of the Childhood Trauma Questionnaire (Bernstein et al., 1997) was our previously validated (Juhasz et al., 2011) tool to measure CHA. The four items of the questionnaire relate to emotional or physical abuse and neglect. It was completed with an additional question about potential loss of parents during childhood. RLE were measured with the validated List of Threatening Experiences questionnaire (Brugha et al., 1985). Financial hardship (FINANC) was derived from the background questionnaire and was previously used to test gene × environmental interaction effect in a study of ours (Sarginson et al., 2014). It captures a personal feeling of financial status (instead of a direct question about income). The original FINANC variable contained five categories, but three categories of FINANC were used for the primary analysis (combining the first two and the last two categories) to gain more appropriate sample sizes: (1) “*living very/quite comfortably*”; (2) “*just getting by*”; and (3) “*finding it difficult to make ends meet/not able to make ends meet*.”

### Genotyping

Genomic DNA was derived from buccal mucosa cells (Juhasz et al., 2009). After extraction of DNA the Sequenom^®^ MassARRAY technology (Sequenom^®^, San Diego, CA, USA^1^) was used to normalize and genotype the samples.

Rs10462028 SNP was selected as a haplotype tag of the 3′-UTR region of the *CLOCK* gene (Haploview^2^). Genotyping was performed according to the ISO 9001:2000 requirements and kept blinded with regard to phenotypic data.

The minor allele frequencies of the selected single nucleotide polymorphism (SNP) can be seen in Table 1B.

**Table 1:** Details of the investigated samples and statistical results of the comparison between Manchester and Budapest subsamples.

	Total sample	Manchester	Budapest	Difference (Manchester vs. Budapest)
**A. Phenotypic description**				
**Demographics**				
Participant number (*n*)	2,157	1,277	880	
Female (*n*, %)	1,503 (69.7%)	893 (69.9%)	610 (69.3%)	*χ*^2^ = 0.092, *p* = 0.761
Age (mean ± SEM)	32.9 (±0.22)	34.02 (±0.29)	31.3 (±0.36)	**T = −5.982, *p* < 0.0001**
**Migraine status**				
migraineID (*n*, %)	600 (27.8%)	399 (31.2%)	201 (22.8%)	***χ*^2^ = 18.326, *p* < 0.0001**
**Depression**				
Reported lifetime depression (*n*, %)	907 (42%)	713 (55.8%)	194 (22%)	***χ*^2^ = 244.087, *p* < 0.0001**
**Bipolar disorder**				
Reported lifetime bipolar disorder (*n*, %)	69 (3.2%)	51 (4%)	18 (2%)	***χ*^2^ = 6.386, *p* = 0.012**
**Stress factors**				
Recent negative life events categories (*n*, %)				
No or mild	1,435 (66.5%)	821 (64.3%)	614 (69.8%)	***χ*^2^ = 14.654, *p* < 0.0001**
Moderate	400 (18.5%)	237 (18.6%)	163 (18.5%)
Severe	318 (14.7%)	219 (17.1%)	99 (11.3%)
Missing data	4 (0.2%)	-	4 (0.5%)
Childhood adversity categories (*n*, %)				
No or mild	1,398 (64.8%)	780 (61.1%)	618 (70.2%)	***χ*^2^ = 29.375, *p* < 0.0001**
Moderate	394 (18.3%)	238 (18.6%)	156 (17.7%)
Severe	355 (16.5%)	254 (19.9%)	101 (11.5%)
Missing data	10 (0.5%)	5 (0.4%)	5 (0.6%)
Financial hardship categories (*n*, %)				
Living very/quite comfortably	1,267 (58.7%)	689 (54%)	578 (65.7%)	***χ*^2^ = 50.215, *p* < 0.0001**
Just getting by	656 (30.4%)	409 (32%)	247 (28.1%)
Finding it difficult/not able to make ends meet	225 (10.4%)	177 (13.9%)	48 (5.5%)
Missing data	9 (0.4%)	2 (0.2%)	7 (0.8%)
**B. Genetic data**				
Minor allele frequencies (MAF)				
rs10462028 (A/G)	33.01%	33.27%	32.66%

*Table 1A shows the phenotypic description of our sample and the statistical results of the comparison between subsamples while Table 1B summarizes data of the genetic variable. migraineID: positive for migraine, data derived from the ID-Migraine questionnaire. SEM, standard error of mean. Childhood adversity categories: (1) *no or mild* (score: 0–3); (2) *moderate* (score: 4–6); and (3) *severe* (score: 7 or more). Recent negative life events categories: (1) *no or mild* (score: 0 or 1); (2) *moderate* (score: 2); and (3) *severe* (score: 3 or more). χ^2^: Pearson χ^2^; p: significance—bold: significant effects*.

### Functional Prediction of rs10462028

According to the 1,000 Genomes database^3^ rs10462028 is in high linkage disequilibrium (LD; *r*^2^ > 0.8) with 70 SNPs covering a region of 132,043 base pairs (from base position 56,288,743 to 56,420,786).

The investigated SNP potentially affects microRNA (miRNA) binding—therefore here, we aim to predict the possible effects of rs10462028 on miRNA-binding and regulation of the *CLOCK* gene. Thus, we have identified miRNAs with seed regions containing rs10462028, and one of the alternative alleles possibly alters the binding affinity. Further miRNA binding sites were predicted and examined near the SNP (as the altered mRNA sequence/structure can affect the accessibility of the region) and additionally around rs1801260 polymorphism that is in high LD (*r^2^* = 0.9, in the same LD-block—according to the 1,000 Genomes database; and *r^2^* = 0.802 in the CEU population according to SNP Function Prediction)^4^ with rs10462028. Rs1801260 is a frequently examined SNP of *CLOCK* because of its proposed effect on activity, sleep onset, and sleep quantity (Katzenberg et al., 1998; Mishima et al., 2005; Benedetti et al., 2007).

The sequence of the *Homo sapiens* CLOCK 3-UTR (NM_001267843.1) transcript variant was obtained from the Nucleotide database of NCBI^5^. SNPs in LD with rs10462028 and rs1801260 have been collected using NIH SNP Function Prediction^4^ with the following parameters: LD ≥0.8 in Population CEU, based on Genotype Data from HapMap CEU, based on Genotype Data from dbSNP: European. We only included 3′-UTR polymorphisms in our further investigation and focused on the *CLOCK* gene in this study.

MiRNA binding sites around polymorphisms were predicted using TargetScan^6^ (Lewis et al., 2003), miRanda predictions on NIH SNP Function Prediction, and MicroSNiPer^7^ (Barenboim et al., 2010). Conserved and poorly conserved sites for miRNA families were both examined. We used genecards.org/ website and miRiAD^8^ (Hinske et al., 2010) database to include only miRNAs with known expression in the CNS by both sources. MiRNA-binding predictions were performed with both the reference and alternative alleles of the polymorphism.

### Statistical Analysis

To calculate Hardy-Weinberg equilibrium (HWE) *p* and LD *r^2^*-values, to run logistic and linear regression analysis with additive, dominant, and recessive genetic models, PLINK v1.07^9^ analysis program was used.

In the *main analyses* we tested the main effect of rs10462028, then the SNP × stress interactions with each of the stress factors (CHA, RLE, FINANC) on migraineID in the total sample. Age and gender were covariates in all analyses. To handle possible ancestral differences, subjects with European white origin (determined by self-reported data derived from the Background questionnaire) were included in the analysis, and population (study sites) was a covariate in those analysis where combined samples were used. To assess our nominally significant findings we used three methods: (a) the gold standard Bonferroni correction to adjust *p-values* for multiple testing in our 12 main analyses [additive, dominant and recessive models of *CLOCK* SNP main effects and SNP × stress factor (CHA, RLE, or FINANC) interactions on migraineID in the total sample] with a Bonferroni-corrected threshold of *p* ≤ 0.004 (0.05/12); (b) as Bonferroni correction is overly conservative and does not take into account the interdependences between the three genetic models of the same SNP, we used another more lenient corrected *p-value* taking into account our four hypotheses *p* ≤ 0.0125 (0.05/4); and (c) finally effects were considered statistically significant in case of reaching a significance value below 0.05 not only in the total sample but also in the two subsamples (Budapest and Manchester separately).

In case of significant results we also ran *additional analyses*. Lifetime bipolar disorder (MANIC) and lifetime depression (DEPR) were added separately to models as covariates—to control for their potential confounding effect.

Furthermore, we tested the main effects of the *CLOCK* SNP on the stress factors (FINANC, CHA, RLE), MANIC, and DEPR—to recognize its potential effect on these components that may contribute to the connection between the SNP and migraine.

For displaying purposes, we calculated positive likelihood ratios (LR+) by dividing the frequency of migraine cases (symptom carriers) by the frequency of control subjects (non-carriers) in each category of each stress variable which showed significant interaction effects with the SNP. Further statistical analyses were performed with IBM SPSS Statistics 23. All statistical testing adopted a two-tailed *p* = 0.05 threshold. As *post hoc* tests, we measured the association of migraine frequencies and the SNP in the categories of stress factors with chi-squared test. To measure differences between our two subsamples in the phenotypic data, we used chi-squared test or independent sample *t*-test. Logistic regression models (with covariates: gender, age, population) were used to replicate the previously identified main effect of the measured stress factors on migraine.

Quanto 1.2 version^10^ was used to calculate the statistical power of our study. Assuming a case-control design (three controls/case) and an additive genetic model with a minor allele frequency between 32 and 33% in our study (*n* = 2,157), we have 96% power to detect genetic main effects associated with a 1.3 odds ratio (OR) for a disease and 80% power to detect a gene × environment interaction (*p* = 0.05 two-tailed) that is associated with 1.5 OR for a disease.

## Results

### Sample

A detailed description of our study sample is described in Table 1A. Females represented approximately 70% of our sample, and the average age (±SEM) was 32.9 (±0.22) years. 27.8% of the participants reported two or three symptoms of migraine type headache based on the ID-Migraine questionnaire and were assigned migraineID. There were significant differences in each phenotypic variable except gender between the two subsamples. Subjects from Manchester showed higher age and higher prevalence of migraine headache, lifetime depression, lifetime bipolar disorder in comparison with the Budapest subsample. They also represented higher percentages in more stressful categories regarding every stress factors (CHA, RLE, FINANC). For detailed results see Table 1A.

### *CLOCK* rs10462028

Rs10462028 was in HWE in the total sample (*p* = 0.36) and in both subsamples (in Manchester: *p* = 0.57; in Budapest: *p* = 0.6).

### Main Effects of Stress Factors on MigraineID

All three stress factors (CHA, RLE, FINANC) showed highly significant (*p* < 0.0001) main effects on migraineID with more severe stress increasing the prevalence of migraineID. For detailed results see Supplementary Table S1. These results legitimize the use of the measured stress factors in our study.

### Main Effect of *CLOCK* rs10462028 on MigraineID

The SNP showed no significant main effect on migraineID. For detailed results see Table 2.

**Table 2:** Statistical results of main genetic effects and interaction effects between rs10462028 and stress factors on migraineID in the total sample.

			Total sample			
**Main effect**						
SNP	A1	Model	OR	L95	U95	*p*
rs10462028	A	ADD	1.109	0.962	1.278	0.152
		DOM	1.144	0.942	1.389	0.175
		REC	1.146	0.854	1.538	0.364
**Interaction**						
SNP × CHA		Model	OR	L95	U95	*p*
		ADD	1.011	0.971	1.053	0.589
		DOM	1.026	0.971	1.084	0.363
		REC	0.988	0.909	1.075	0.784
SNP × RLE		Model	OR	L95	U95	*p*
		ADD	0.942	0.849	1.044	0.255
		DOM	0.930	0.805	1.076	0.332
		REC	0.908	0.738	1.117	0.361
SNP × FINANC		Model	OR	L95	U95	*p*
		ADD	**0.779**	**0.634**	**0.956**	**0.017**
		DOM	0.815	0.617	1.075	0.148
		REC	**0.54**	**0.348**	**0.836**	**0.006**

*Table 2 shows the statistical results of main genetic effects of *CLOCK* rs10462028 and interaction effects between the SNP and stress factors on migraineID in the total sample. SNP, single nucleotide polymorphism; A1, minor (and effect) allele; CHA, childhood adversity; RLE, recent negative life events; FINANC, financial hardship; OR, odds ratio; L95-U95, 95% confidence interval; ADD, additive model; DOM, dominant model; REC, recessive model; p, significance—bold: significant effects. Covariates in the model: age, gender and population*.

### Rs10462028 × Stress Factors Interaction on MigraineID

The investigated SNP was tested in separate analyses in interaction with the measured stress factors on migraineID in the total sample. Results of the regression models are summarized in Table 2.

The SNP showed no significant interaction either with CHA or with RLE.

There was a significant interaction effect between SNP and FINANC on migraineID in the total sample (in additive and recessive models).

**Figure 2:**
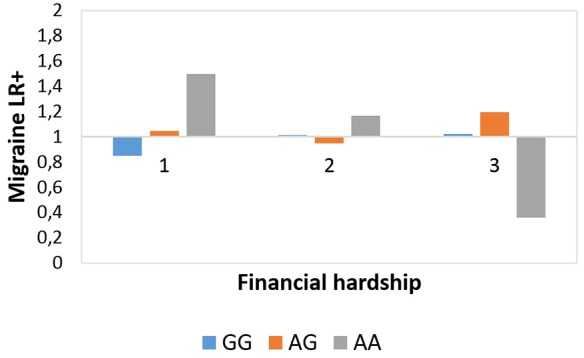
Rs10462028 × financial hardship effect on migraineID. The interaction effect between *CLOCK* rs10462028 and financial hardship on migraineID is shown. FINANC, financial hardship; LR+, positive likelihood ratio. Number of cases in FINANC_1 (*living very/quite comfortably*) group: GG = 575, AG = 550, AA = 142. Number of cases in FINANC_2 (*just getting by*) group: GG = 294, AG = 282, AA = 80. Number of cases in FINANC_3 (*finding it difficult/not able to make ends meet*) group: GG = 102, AG = 100, AA = 23.

Figure 2 shows that subjects with AA genotype have lower degree of migraine LR+ at the most severe but a higher degree of migraine LR+ in the least severe financial hardship category. *Post hoc* chi-squared tests validated that at the most severe FINANC category there was a significantly lower frequency of migraineID among those with AA genotype vs. GG (*χ*^2^ = 3.916, *p* = 0.048) and AG (*χ*^2^ = 5.259, *p* = 0.022) genotypes. In addition, at the *living very/quite comfortably* level of financial difficulties those with AA genotype showed significantly higher migraineID frequency vs. GG genotype (*χ*^2^ = 7.279, *p* = 0.007) and vs. AG genotype at a tendency level (*χ*^2^ = 2.98, *p* = 0.084).

**Figure 3:**
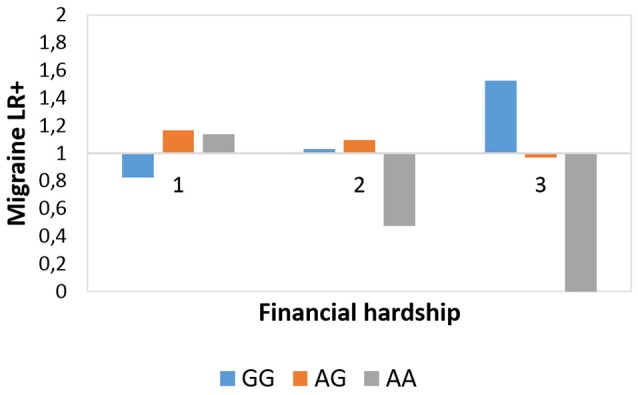
Rs10462028 × financial hardship effect on migraineID in the Budapest subsample. The interaction effect between CLOCK rs10462028 and financial hardship on migraineID in the Budapest subsample is shown. FINANC, financial hardship; LR+, positive likelihood ratio. Number of cases in FINANC_1 (*living very/quite comfortably*) group: GG = 269, AG = 242, AA = 67. Number of cases in FINANC_2 (*just getting by*) group: GG = 106, AG = 117, AA = 24. Number of cases in FINANC_3 (*finding it difficult/not able to make ends meet*) group: GG = 23, AG = 19, AA = 6.

**Figure 4:**
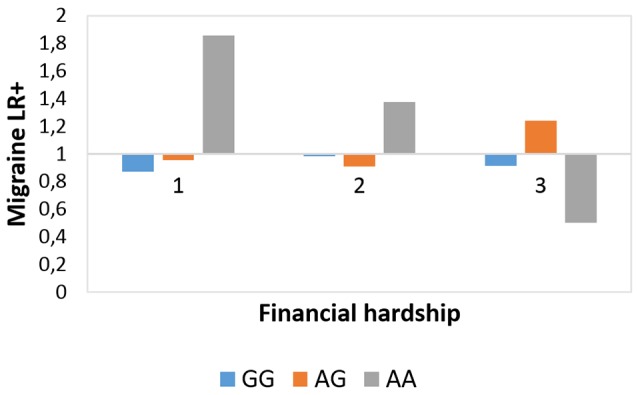
Rs10462028 × financial hardship effect on migraineID in the Manchester subsample. The interaction effect between *CLOCK* rs10462028 and financial hardship on migraineID in the Manchester subsample is shown. FINANC, financial hardship; LR+, positive likelihood ratio. Number of cases in FINANC_1 (*living very/quite comfortably*) group: GG = 306, AG = 308, AA = 75. Number of cases in FINANC_2 (*just getting by*) group: GG = 188, AG = 165, AA = 56. Number of cases in FINANC_3 (*finding it difficult/not able to make ends meet*) group: GG = 79, AG = 81, AA = 17.

The interaction effect between rs10462028 and FINANC on migraineID was also tested in both subsamples (see Table 3). In Budapest both significant results were replicated (Figure 3). However, in Manchester only the recessive model showed a significant effect, and the additive model was not significant (Figure 4). So, only the recessive model was significant in all three samples—our main result with a *p* = 0.006 (see Table 2) is nominally significant, but only a trend effect was reached after Bonferroni-correction. However, considering the interdependence of the genetic models, it would survive the corrected significance threshold when only different hypotheses were corrected.

**Table 3:** Statistical results of interaction effects between rs10462028 and financial hardship on migraineID in the subsamples.

Interaction		Manchester				Budapest			
SNP × FINANC	Model	OR	L95	U95	*p*	OR	L95	U95	*p*
	ADD	0.811	0.632	1.042	0.101	**0.612**	**0.408**	**0.916**	**0.017**
	DOM	0.9	0.643	1.26	0.539	*0.596*	*0.353*	*1.006*	*0.053*
	REC	**0.525**	**0.312**	**0.88**	**0.015**	**0.279**	**0.083**	**0.939**	**0.039**

*Table 3 shows the statistical results of interaction effects between *CLOCK* rs10462028 and financial hardship on migraineID in the subsamples separately (Manchester and Budapest)—to test the replicability of the significant SNP × FINANC effect found in the total sample. SNP, single nucleotide polymorphism; FINANC, financial hardship; OR, odds ratio; L95-U95, 95% confidence interval; ADD, additive model; DOM, dominant model; REC, recessive model; p, significance—bold: significant effects, italic: trend effects. Covariates in the model: age, gender*.

When MANIC and DEPR were added separately to the models as covariates (to control for their potential effect on migraineID) the SNP × FINANC interaction remained significant both in the additive and recessive models (and not significant in the dominant model)—with OR-values very similar to the original ones (for results see Supplementary Table S2).

### Rs10462028 × FINANC Interaction on MigraineID According to Alternative Groupings of FINANC

Because the relatively low number of subjects at the most serious financial hardship level could potentially bias our results, we decided to run additional analyses with a financial difficulties variable that contains only two levels: (1) living very/quite comfortably (*n* = 1,309); and (2) just getting by/finding it difficult or not able to make ends meet (*n* = 904). These analyses also gave us the opportunity to test the differential effects of the SNP at the favorable and at the adverse end of the financial status spectrum that was suggested at the *a priori* level. Significant additive interaction effects between the SNP and financial hardship on migraineID were replicated in the total sample and in the Budapest subsample, but only a tendency was found in the Manchester subsample (Supplementary Table S3). Additionally, the original five-level financial hardship variable was also tested—showing significant interaction effects with the SNP on migraineID in the total sample and in both subsamples with very similar OR-values (Supplementary Table S3).

### Main Effects of rs10462028 on Stress Factors, Bipolar and Unipolar Depression

The SNP showed no significant main effects on any of the measured variables (FINANC, CHA, RLE, MANIC, and DEPR) in the total sample (for detailed results see Supplementary Table S4). Thus, our significant interaction results are not due to gene-environment correlations.

### *In Silico* Functional Analysis of the Genetic Region Tagged by rs10462028

We found several potential miRNA binding sites around rs10462028 and rs1801260 (in LD with the candidate SNP) with predicted change in binding due to the polymorphisms. The miRNA names, SNP allele effects, and prediction algorithms are summarized in Supplementary Table S5. Significantly predicted miRNAs were: miR-409-5p for rs10462028; miR-365b-3p, miR-365a-3p, and miR-664a-5p for rs1801260.

## Discussion

Our exploratory study demonstrated a connection between a circadian gene variant and migraine: *CLOCK* rs10462028 was associated with a migraine phenotype in interaction with financial difficulties in a European cohort from Manchester and Budapest. Similarly to our results previous meta-analyses of migraine GWASs did not show a significant main effect of *CLOCK* (Anttila et al., 2013; Gormley et al., 2016). However, our integration of a chronic stress factor, specifically financial difficulties, pointed out the involvement of rs10462028 in migraine. Interestingly, childhood stress or RLE have not shown the same effect. Our results emphasize the importance to involve different stressors to identify genetic vulnerability to migraine.

### Rs10462028 Selectively Interacts With Financial Hardship but Not Childhood Adversity and Recent Negative Events in Migraine

Circadian rhythms are organized on a daily basis, and stressors might express their influences through different pathways based on their genesis and timing. CHA represents negative effects from the earlier past, while the other two stressors are recent ones. RLE might have a better chance to contribute to the disturbance of circadian rhythms, but they are temporary situations, and that seems to be a significant difference from financial hardship. It is also a much diverse construct (in contrast to financial difficulties) representing multiple stressors.

Financial hardship is a variable of chronic stress—a proxy for deprivations and social difficulties (Sarginson et al., 2014). Its effect is reminiscent of the chronic mild stress model often used in animal studies of depression (Willner, 2017). Both situations can cause a permanent feeling of vulnerability and insecurity. Socioeconomic inequalities are major contributors to multimorbidity and mortality according to a prospective longitudinal study (Katikireddi et al., 2017), and even perceived financial strain which can be experienced at any level of socioeconomic status has been connected to earlier disability and increased mortality (Epel et al., 2018). Previously, our research group presented that different financial difficulties’ variables showed a specific interacting role with polymorphisms of *NOS1* (Sarginson et al., 2014) and *5-HTTLPR* (Gonda et al., 2016) to affect depression, similarly as rs10462028 of *CLOCK* affected migraineID in the present study. Interestingly, our identified SNP × financial difficulties interaction seems to affect specifically migraine—our results were not confounded by lifetime bipolar and unipolar depression. Furthermore, we were able to replicate our main result in both subsamples despite significant differences in phenotypic data.

A previous study (Alstadhaug et al., 2008) suggested that social, work-related stress, and restorative effects of sleep might have a bigger role in the temporal patterns of migraine attacks than the actual biological circadian rhythms. However, other authors (Gori et al., 2005) highlighted that a desynchronization between the circadian clock mechanism and lifestyle might contribute to stress and migraine severity. According to our results, chronic stress and the circadian clock may interact in migraine pathophysiology.

### Crossover Interaction Between rs10462028 and Financial Hardship

Our results suggest a crossover interaction between rs10462028 and financial difficulties on migraine: AA genotype seems to be a risk factor at a more favorable financial status and a protective factor at a more adverse financial stress level. This crossover type of interaction between genes and environmental factors in the background of diseases is not unprecedented. A review (Belsky et al., 2009) that discusses the environmental sensitivity hypothesis suggested that in gene × environment studies, instead of simply looking for genetic vulnerability, we should see these genetic variants as they make individuals more susceptible to both positive and negative environmental situations. This perspective can explain those results when the same genes seem to cause vulnerability to adversity and simultaneously promote an advantage in the lack of adversity—including evidence showing crossover interactions between different environmental effects on psychopathologies and *MAOA*, *5-HTTLPR*, and *DRD4* genes (Belsky et al., 2009). Our research group previously found that a variant of interleukin-1*β* gene (*IL1β*) had protective effect against depressive symptoms at a low degree of stressors, but it was a risk factor at high stress exposure (Kovacs et al., 2016). So, rs10462028 could also represent a so-called “plasticity gene” effect (Belsky et al., 2009) instead of a simple “vulnerability gene” effect.

In this kind of interaction the effects could statistically “obliterate” each other if we only test the main effect of the gene. Consequently, we should not expect to see these genetic effects in GWASs. These considerations highlight the importance of measuring the impact of relevant stress factors in genetic studies. Gene × environment studies can provide significant contribution to the “missing heritability” found in GWASs (Manolio et al., 2009; Juhasz et al., 2014).

### Molecular Biology and Function of rs10462028

As we showed, rs10462028 (and rs1801260 in LD with it) affects miRNA binding. The most relevant predicted miRNAs have been mostly connected to different cancer types (Wang et al., 2013; Geng et al., 2016; Sahin et al., 2017; Yu et al., 2017). Migraine has not been associated with these miRNAs; the presence of chronic stress, however, might trigger this connection. Circadian genes might have an important role in cancer development through the disruption of circadian control of energy balance, immune function, and aging (Fu and Kettner, 2013). Similarly, these processes lead to functional decline in the brain (Kondratova and Kondratov, 2012). A recent study also suggested that *CLOCK* regulates brain plasticity during key developmental periods (Kobayashi et al., 2015) and thus may contribute to several brain disorders, such as migraine. However, our study did not confirm a possible interaction effect between *CLOCK* and CHA on migraine.

Functional polymorphisms in miRNAs or in their target sequences may alter regulation of gene expression (Mishra et al., 2008). Several miRNAs appear to regulate neural protein expressions, and various diseases have been connected to the altered translational regulation by the miRNA system (Dwivedi, 2011). Evidence also suggests that miRNAs are significant factors in circadian clock timing, presenting novel therapeutic targets for diseases related to circadian rhythms (Hansen et al., 2011). MiRNAs are also important in mediating environmental effects that modify gene expression (Lopizzo et al., 2015).

Our results showed allele effects impairing miRNA binding which can contribute to higher production of CLOCK protein—based on the assumption that weakened mRNA-miRNA interactions can increase protein levels (Moszyńska et al., 2017). Higher level of CLOCK can contribute to an extensive disturbance in the main circadian pacemaker because of the strong interactions inside and between transcriptional/post-translation feedback loops. This confusion at the SCN may lead to a higher susceptibility for environmental effects which might explain the crossover interaction we found. A desynchronization between lifestyle and the circadian clock caused by financial difficulties may enhance the described process—finally leading to migraine susceptibility. In the case of *CLOCK* variants the C allele (in Supplementary Table S5 the G allele on the complementary strand) of rs1801260 was associated with eveningness, delayed sleep onset, and reduced sleep (Katzenberg et al., 1998; Mishima et al., 2005; Benedetti et al., 2007), and this allele is in linkage with rs10462028 A allele^11^. Thus, eveningness, delayed sleep onset, and reduced sleep might be advantageous during financial hardship to prevent migraine type headaches while disadvantageous during financial stability. These assumptions are hypothetical at the moment and need to be tested in future studies, but they could provide a pathway that integrates chronic stress, the circadian clock, and potential epigenetic processes in migraine pathophysiology.

## Limitations

The main advantage of our study: the use of detailed phenotypic data allowed us to identify a specific gene × stressor interaction which might be relevant in migraine. However, our work has some limitations. We demonstrated a relationship between a circadian gene and migraine for the first time in a relatively small sample. Nevertheless, power analyses revealed enough power to detect the investigated genetic effects. Our main result was nominally significant, but only a trend effect was reached after the gold standard Bonferroni correction of multiple testing—therefore, our findings should be interpreted with caution and independent replications are needed to confirm our results. We used a cross-sectional design, therefore, the causative role of stressors and the temporal relationship between stress and migraine could not be investigated. Our migraine screening tool was a short questionnaire which did not provide proper medical diagnosis although it is a widely used method (Anttila et al., 2013). The ID-migraine questionnaire does not provide information regarding migraine subtypes (for example migraine with and without aura). Future studies focusing on possible differences in the role of circadian rhythmicity between migraine subtypes are needed. Nearly 70% of our sample consisted of females who are affected by migraine three times more often than men (Goadsby et al., 2017). Female gender is also a risk factor for sleeping problems, including insomnia and obstructive sleep apnea (Rains, 2018), and sex differences in circadian timing systems may be important in circadian-related diseases (for a review see Bailey and Silver, 2014). Future studies involving more male subjects are needed to gain knowledge about the possible role of gender in the association of migraine and circadian mechanisms. Finally, we need to mention that the circadian clock mechanism is far more complex—we only focused on one variant of one circadian gene. With this strategy we were able to identify a possible role of the circadian mechanism in the pathophysiology of migraine which suggests a need to further investigate the connection of circadian rhythms and migraine in a broader context.

## Conclusions

In conclusion, our results show that genetic variation in the *CLOCK* gene is associated with migraine depending on the level of perceived financial stress. This could be explained most likely through a process where persistent stress disturbs the physiological function of the circadian clock mechanism, possibly *via* epigenetic regulation, specifically miRNA binding.

## Data Availability Statement

The datasets generated for this study are available on request to the corresponding author.

## Ethics Statement

Our study was approved by local Ethics Committees (Scientific and Research Ethics Committee of the Medical Research Council, Budapest, Hungary, ad.225/KO/2005; ad.323-60/2005-1018EKU and ad.226/KO/2005; ad.323-61/2005-1018 EKU; North Manchester Local Research Ethics Committee, Manchester, UK REC reference number: 05/Q1406/26) and was carried out in accordance with the Declaration of Helsinki. All participants provided their written informed consent to participate in this study.

## Author Contributions

GB, GJ, and JD designed the study and wrote the protocol. DB undertook the statistical analysis and managed the literature search. DB, XG, NE, PP, VA, and LK wrote the first draft of the manuscript. All authors contributed to and have approved the final manuscript.

## Conflict of Interest

Preliminary data from this study were presented at the following events *Conference of Hungarian Clinical Neurogenetic Society*, 1–2 December 2017, Kecskemet, Hungary (lecture); *30th ECNP Congress*, 2–5 September 2017, Paris, France (poster presentation); *13th World Congress of Biological Psychiatry*, 18–22 June 2017, Copenhagen, Denmark (poster presentation); *24th Congress of the Hungarian Headache Society*, 5–6 May 2017, Siofok, Hungary (lecture); *XXVI World Congress of Psychiatric Genetics*, 11–15 October 2018, Glasgow, Scotland (poster presentation); *Semmelweis University PhD Scientific Days*, 25–26 April 2019, Budapest, Hungary (poster presentation). JD variously performed consultancy, speaking engagements and research for Bristol-Myers Squibb, AstraZeneca, Eli Lilly, Schering Plough, Janssen-Cilag and Servier (all fees are paid to the University of Manchester to reimburse them for the time taken); he also has share options in P1vital. The remaining authors declare that the research was conducted in the absence of any commercial or financial relationships that could be construed as a potential conflict of interest.

## Footnotes

^1^https://www.gene-quantification.de/sequenom/

^2^https://www.broadinstitute.org/haploview/haploview

^3^http://www.internationalgenome.org/data

^4^https://snpinfo.niehs.nih.gov/snpinfo/snpfunc.html

^5^https://www.ncbi.nlm.nih.gov/nucleotide/

^6^http://www.targetscan.org

^7^http://vm24141.virt.gwdg.de/services/microsniper/

^8^https://bmi.ana.med.uni-muenchen.de/miriad/downloads

^9^http://pngu.mgh.harvard.edu/purcell/plink/

^10^https://preventivemedicine.usc.edu/download-quanto/

^11^https://ldlink.nci.nih.gov/?tab=ldhap
