# Supplementary material for: Financial Stress Interacts With CLOCK Gene to Affect Migraine
Source: Front Behav Neurosci. 2020 Jan 24;13:284. doi: 10.3389/fnbeh.2019.00284 (PMC6993567; doi:10.3389/fnbeh.2019.00284)
Supplement: Supplementary file 1 [file Data_Sheet_1.pdf]

Supplementary Material

Supplementary Table 1. Statistical results of main effects of stress factors on migraineID

	**Total sample**			
**Main effect on migraineID**				
	Beta	L95	U95	p
CHA	0.069	1.043	1.101	<0.0001
RLE	0.245	1.189	1.373	<0.0001
FINANC (3 categories)	0.345	1.233	1.617	<0.0001
FINANC (2 categories)	0.58	1.467	2.172	<0.0001
FINANC (5 categories)	0.284	1.186	1.489	<0.0001

Supplementary Table 1 shows the statistical results of additional analyses of main effects of stress factors (CHA, RLE, FINANC with 3, 2 and 5 categories) on migraineID in the total sample. L95-U95: 95% confidence interval; p: significance; CHA: childhood adversity; RLE: recent negative life events; FINANC: financial hardship. Covariates in the model: age, gender and population.

Supplementary Table 2. Statistical results of interaction effects between rs10462028 and financial hardship on migraineID with controlling for bipolar and unipolar lifetime depression

Total sample

**Interaction**						
		**MANIC as added covariate**				
SNP x FINANC	A1	Model	OR	L95	U95	p
	A	ADD	**0.776**	**0.632**	**0.953**	**0.015**
		DOM	0.81	0.614	1.069	0.137
		REC	**0.539**	**0.347**	**0.835**	**0.006**
		**DEPR as added covariate**				
SNP x FINANC	A1	Model	OR	L95	U95	p
	A	ADD	**0.774**	**0.629**	**0.952**	**0.015**
		DOM	0.81	0.612	1.072	0.141
		REC	**0.532**	**0.342**	**0.827**	**0.005**

Supplementary Table 2 shows the statistical results of interaction effects between *CLOCK* rs10462028 and financial hardship on migraineID – with lifetime bipolar disorder and lifetime depression added separately as covariates to the model to control for their potential effect. SNP: single nucleotide polymorphism; FINANC: financial hardship; MANIC: lifetime bipolar disorder; DEPR: lifetime depression; A1: minor (and effect) allele; OR: odds ratio; L95-U95: 95% confidence interval; ADD: additive model; DOM: dominant model; REC: recessive model; p: significance – bold: significant effects. Covariates in the model: age, gender, population and lifetime depression.

Supplementary Table 3. Statistical results of the additional analyses of interaction effects between rs10462028 and two- and five-level financial hardship on migraineID

**Interaction with two-level financial hardship**			**Total sample**				**Manchester**				**Budapest**			
SNP	A1	Model	OR	L95	U95	p	OR	L95	U95	p	OR	L95	U95	p
rs10462028	A	ADD	**0.737**	**0.553**	**0.981**	**0.037**	0.758	0.528	1.088	0.133	**0.592**	**0.354**	**0.989**	**0.045**
		DOM	0.727	0.49	1.078	0.113	0.806	0.489	1.329	0.398	*0.569*	*0.292*	*1.112*	*0.099*
		REC	*0.557*	*0.308*	*1.01*	*0.054*	*0.517*	*0.249*	*1.069*	*0.075*	*0.29*	*0.074*	*1.143*	*0.077*

**Interaction with five-level financial hardship**			**Total sample**				**Manchester**				**Budapest**			
SNP	A1	Model	OR	L95	U95	p	OR	L95	U95	p	OR	L95	U95	p
rs10462028	A	ADD	**0.787**	**0.662**	**0.936**	**0.007**	*0.829*	*0.674*	*1.02*	*0.076*	**0.604**	**0.424**	**0.86**	**0.005**
		DOM	0.832	0.656	1.054	0.128	0.907	0.685	1.202	0.499	**0.611**	**0.383**	**0.977**	**0.04**
		REC	**0.546**	**0.38**	**0.786**	**0.001**	**0.574**	**0.377**	**0.873**	**0.009**	**0.225**	**0.079**	**0.638**	**0.005**

Supplementary Table 3 shows the statistical results of the additional analyses of interaction effects between *CLOCK* rs10462028 and two- and five-level financial hardship on possible migraine. SNP: single nucleotide polymorphism; A1: minor (and effect) allele; OR: odds ratio; L95-U95: 95% confidence interval; ADD: additive model; DOM: dominant model; REC: recessive model; p: significance – bold: significant effects, italic: trend effects. Covariates in the model: age, gender and population in total sample; age and gender in subsamples.

Supplementary Table 4. Statistical results of main effects of rs10462028 on measured stress factors, lifetime bipolar and unipolar depression

		**Total sample**				
**Main effect**		**On CHA**				
SNP	A1	Model	Beta	L95	U95	p
rs10462028	A	ADD	-0.013	-0.221	0.194	0.900
		DOM	0.019	-0.261	0.299	0.892
		REC	-0.107	-0.547	0.333	0.633
		**On RLE**				
SNP	A1	Model	Beta	L95	U95	p
rs10462028	A	ADD	-0.038	-0.119	0.043	0.359
		DOM	-0.053	-0.162	0.056	0.341
		REC	-0.039	-0.210	0.132	0.655
		**On FINANC**				
SNP	A1	Model	Beta	L95	U95	p
rs10462028	A	ADD	-0.002	-0.044	0.040	0.920
		DOM	-1.211^{-5}	-0.057	0.057	0.999
		REC	-0.009	-0.099	0.079	0.832
		**On MANIC**				
SNP	A1	Model	OR	L95	U95	p
rs10462028	A	ADD	0.923	0.642	1.327	0.667
		DOM	1.032	0.636	1.674	0.899

		REC	0.597	0.237	1.501	0.273
		**On DEPR**				
SNP	A1	Model	OR	L95	U95	p
rs10462028	A	ADD	0.971	0.846	1.114	0.676
		DOM	0.920	0.765	1.108	0.380
		REC	1.076	0.805	1.437	0.622

Supplementary Table 4 shows the statistical results of additional analyses of main effects of *CLOCK* rs10462028 on measured stress factors (CHA, RLE, FINANC), lifetime bipolar (MANIC) and unipolar depression (DEPR) in the total sample. SNP: single nucleotide polymorphism; A1: minor (and effect) allele; OR: odds ratio; L95-U95: 95% confidence interval; ADD: additive model; DOM: dominant model; REC: recessive model; p: significance; CHA: childhood adversity; RLE: recent negative life events; FINANC: financial hardship; MANIC: lifetime bipolar disorder; DEPR: lifetime depression. Covariates in the model: age, gender and population.

Supplementary Table 5. Predicted miRNA bindings in the 3'UTR of *CLOCK* gene around rs10462028 and rs1801260 SNPs.

SNP	miRNA name	Allele effect on miRNA binding	Prediction algorithm
**rs10462028 (A/G)**	miR-4640-3p	#	TargetScan
	miR-2116-3p	#	TargetScan
	miR-323b-3p	#	TargetScan
	**miR-409-5p**	**A ↓**	microSNiPer
	miR-4532	A ↓#	microSNiPer
	miR-106b-3p	A ↓#	microSNiPer
**rs1801260 (G/A)**	**miR-365b-3p**	**G↓**	microSNiPer
	**miR-365a-3p**	**G↓**	microSNiPer
	**miR-664a-5p**	**G↓**	microSNiPer
	miR-6504-5p	A↓#	microSNiPer
	miR-1914-5p	A↓#	microSNiPer
	miR-141-3p	A↓#	miRanda, Targetscan
	miR-200a-3p	A↓#	miRanda, Targetscan
	miR-1225-3p	A↓#	miRanda

Supplementary Table 5 shows the predicted miRNA bindings in the 3' UTR of *CLOCK* gene around rs10462028 and rs1801260 SNPs with the directions of allele effects. ↓: may impair binding and potentially alter protein translation; #: no direct effect on miRNA seed region, or weak change (G-U pair instead of G-C pair). Bold: direct effect on miRNA seed region, or significant change in binding. MiRNA: microRNA; 3' UTR: 3' untranslated region.
